# Automated whole slide image analysis for a translational quantification of liver fibrosis

**DOI:** 10.1038/s41598-022-22902-w

**Published:** 2022-11-04

**Authors:** Cindy Serdjebi, Karine Bertotti, Pinzhu Huang, Guangyan Wei, Disha Skelton-Badlani, Isabelle A. Leclercq, Damien Barbes, Bastien Lepoivre, Yury V. Popov, Yvon Julé

**Affiliations:** 1 Biocellvia, 10 Rue Grignan, 13001 Marseille, France; 2 Division of Gastroenterology, Hepatology and Nutrition, Beth Israel Deaconess Medical Center, Harvard Medical School, 330 Brookline Ave, Boston, MA 02215 USA; 3 Department of Radiation Oncology, The First Affiliated Hospital of Sun Yat-Sen University, 58 Zhongshan 2Nd Rd, Yuexiu District, Guangzhou, Guangdong Province China; 4 Laboratory of Hepato-Gastroenterology, Institut de Recherche Expérimentale Et Clinique, Université Catholique de Louvain (UCLouvain), Avenue Emmanuel Mounier 52, 1200 Brussels, Belgium

**Keywords:** Drug development, Experimental models of disease, Preclinical research, Translational research, Computational science, Software

## Abstract

Current literature highlights the need for precise histological quantitative assessment of fibrosis which cannot be achieved by conventional scoring systems, inherent to their discontinuous values and reader-dependent variability. Here we used an automated image analysis software to measure fibrosis deposition in two relevant preclinical models of liver fibrosis, and established correlation with other quantitative fibrosis descriptors. Longitudinal quantification of liver fibrosis was carried out during progression of post-necrotic (CCl_4_-induced) and metabolic (HF-CDAA feeding) models of chronic liver disease in mice. Whole slide images of picrosirius red-stained liver sections were analyzed using a fully automated, unsupervised software. Fibrosis was characterized by a significant increase of collagen proportionate area (CPA) at weeks 3 (CCl_4_) and 8 (HF-CDAA) with a progressive increase up to week 18 and 24, respectively. CPA was compared to collagen content assessed biochemically by hydroxyproline assay (HYP) and by standard histological staging systems. CPA showed a high correlation with HYP content for CCl_4_ (r = 0.8268) and HF-CDAA (r = 0.6799) models. High correlations were also found with Ishak score or its modified version (r = 0.9705) for CCl_4_ and HF-CDAA (r = 0.9062) as well as with NASH CRN for HF-CDAA (r = 0.7937). Such correlations support the use of automated digital analysis as a reliable tool to evaluate the dynamics of liver fibrosis and efficacy of antifibrotic drug candidates in preclinical models.

## Introduction

Accurate assessment of fibrosis is paramount to determine the severity of chronic liver diseases, such as non-alcoholic steatohepatitis (NASH), as it primarily drives mortality in patients^1–4^. In preclinical studies, fibrosis amount is evaluated from the biochemical assay of hydroxyproline (HYP) content^5–8^ and, in parallel, by means of histological scoring systems which are applied for both preclinical studies and clinical investigations from human biopsy^9,10^. Histological scoring systems typically characterize liver fibrosis localization and pattern due to the ordinal nature of their classification. The amount of fibrosis as such is not measured, potentially limiting clinical interpretation, especially in the context of clinical trials with limited duration, since current clinical histopathology scoring systems might be not sufficiently sensitive to detect small changes in fibrosis pattern. Moreover, scoring fibrosis is reader-dependent and therefore might suffer from intra- and inter-variability^10–12^. Ultimately, histological scoring systems alone cannot be considered as versatile and comprehensive tools to monitor liver fibrosis at early and mildly fibrotic stages, which greatly hampers the assessment of the efficacy of antifibrotic drugs.

Digital pathology has considerably developed over the past years with the following advantages: to provide a continuous measurement of histological endpoints, independency from the reader’s subjectivity. Computerized image techniques were already applied to histological liver section in various fibrotic rodent models^13–15^ and to human biopsies^16–18^. One of the first applications of digital pathology in the field of hepatology was liver fibrosis assessment. Most of the commonly used techniques are based on the computerized segmentation of collagen fibers stained with picrosirius red (PSR) or Masson’s trichrome. Fibrosis is assessed from the quantification of the collagen proportionate area (CPA). However, some limitations apply to most of available computerized image analyses such as: (i) a number of miscellaneous manual interventions are required; these may involve operator-subjectivity; (ii) CPA is determined mainly from selected regions of interest and whole tissue section analysis is not systematically performed, which may lead to bias given the heterogenous distribution of collagen fibers across the lobules and the liver section. To overcome these limitations, we developed an automated computerized image analysis software (MorphoQuant, Biocellvia, France) which allows an accurate, reliable, fast and reproducible analysis of NAFLD/NASH histological features in rodent models and humans regardless of the operator from whole slide images^4,14^. In the present study, this tool was used to quantitatively analysis fibrosis in two commonly used preclinical models of liver fibrosis in mice: the chronic carbon tetrachloride (CCl_4_) model, with prevalent septal fibrosis^19–22^, and the high-fat choline-deficient amino acid (HF-CDAA) dietary mouse model for fibrotic steatohepatitis in which perisinusoidal fibrosis predominates^6,23^. Specifically, the two investigated models received optimized treatments to induce a sustainable liver fibrosis comparable for certain criteria to that observed in human liver diseases^6,20^. In these mouse models, the performances of the automated software on liver fibrosis quantification were investigated and compared to the more conventional techniques, i.e., the relative HYP assay and fibrosis histological scoring systems.

## Results

### Automated CPA quantification captures longitudinal progression of fibrosis in the models

**Figure 1:**
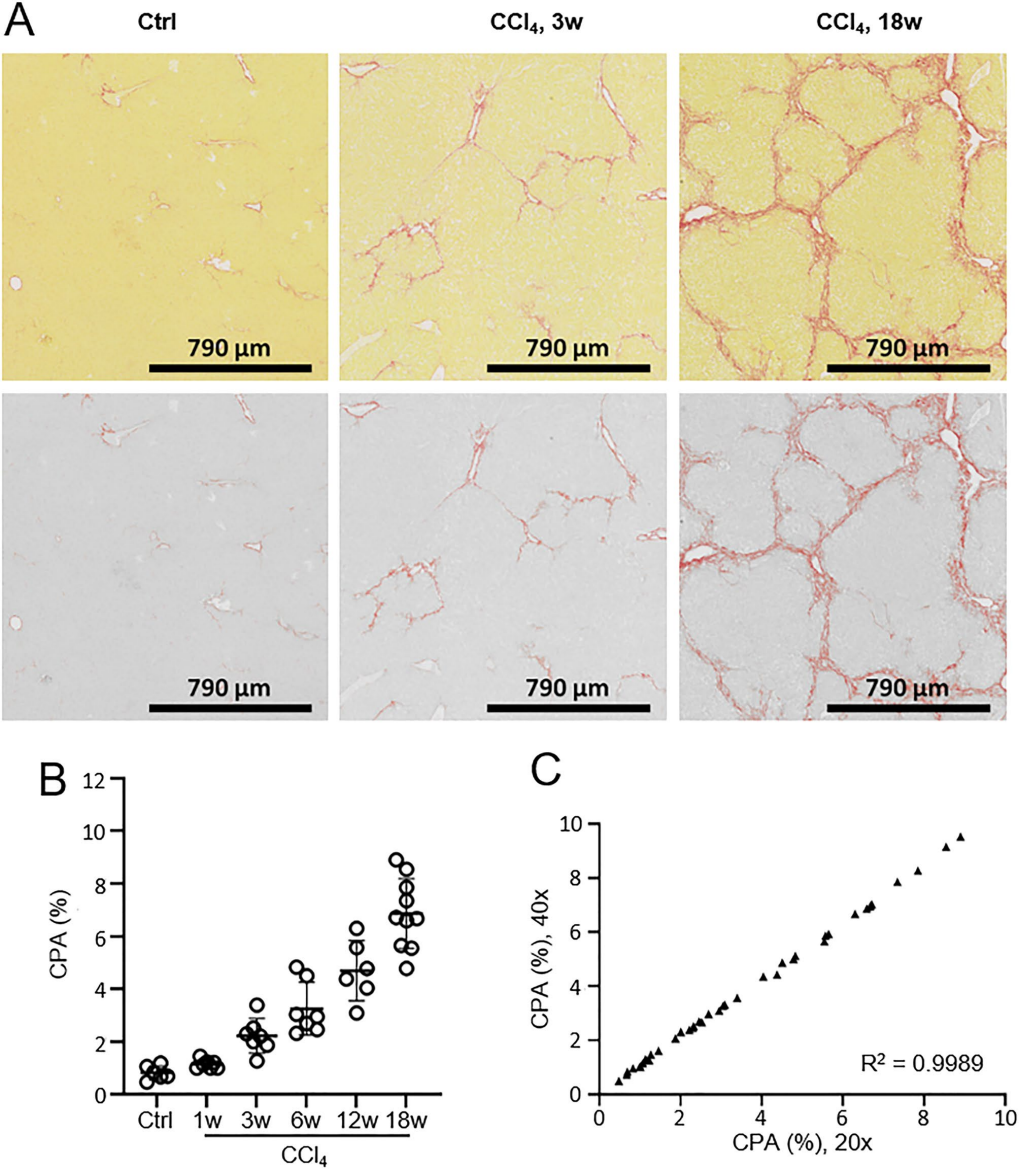
(**A**) Representative images of liver sections from CCl4-treated mice at weeks 0 (Ctrl), 3 (3w) and 18 (18w); the upper panel corresponds to native images of picrosirius red-stained liver sections, the bottom panel shows the corresponding digital images, obtained with MorphoQuant software, in which collagen fibers were pseudocolored in red and liver tissue in grey. **(B)** Evolution of collagen proportionate area (CPA%) relative to the selected timepoints. (**C**) Correlation of CPA values obtained in the same picrosirius red-stained sections at 20 × and 40 × respectively using MorphoQuant software. Results were expressed as mean value ± SD (n = 3 sections/mouse, 6–10 mice/group). R^2^: coefficient of determination. *p* value < 0.05 was considered statistically significant.

**Figure 2:**
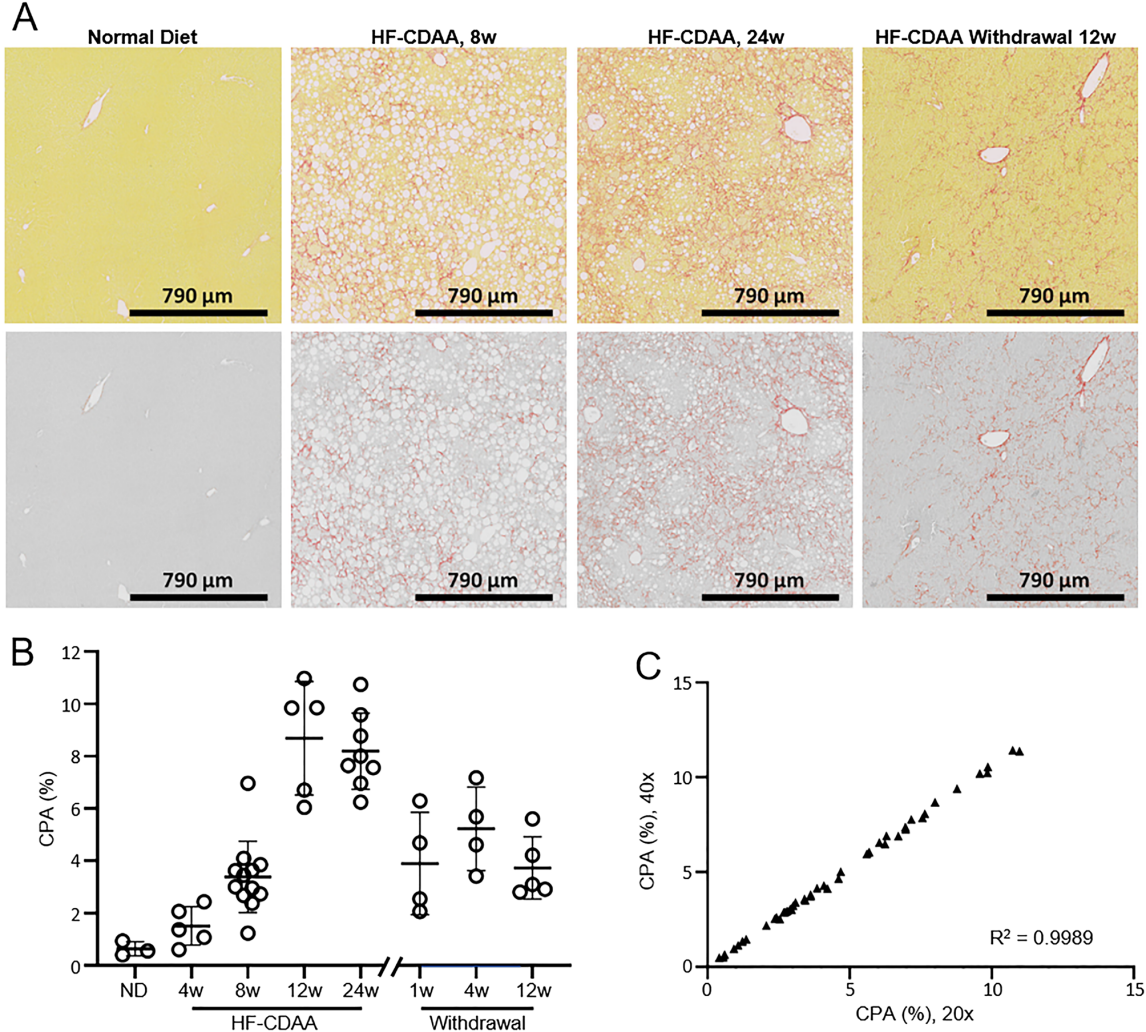
(**A**) Representative images of liver sections from HF-CDAA-treated mice at weeks 0 (normal diet), 8 (8w), 24 (24w) and a recovery period of 12 weeks following 8 weeks HF-CDAA treatment; the upper panel corresponds to native images of picrosirius red-stained liver sections, the bottom panel shows the corresponding digital images, obtained with MorphoQuant software, in which collagen fibers were pseudocolored in red and liver tissue in grey. (**B**) Evolution of collagen proportionate area (CPA%) after HF-CDAA treatment relative to selected time points and after HF-CDAA withdrawal following an 8-week HF-CDAA induction. (**C**) Correlation of CPA values obtained in the same picrosirius red-stained sections at 20 × and 40 × respectively using MorphoQuant software. Results were expressed as mean value ± SD (n = 3 sections/mouse, 6–10 mice/group). R^2^: coefficient of determination. *p* value < 0.05 was considered statistically significant.

Experiments were carried out on two different mouse models, CCl_4_ and HF-CDAA, known to develop fibrosis with substantial differences in fibrosis patterns. In CCl_4_-treated mice, previous observations reported the development of central and septal fibrosis from incipient (week 1), moderate (week 3) or advanced (week 6) up to early cirrhosis (week 12) (Fig. 1A)^20^. In this study, collagen deposition was analyzed longitudinally at weeks 1, 3, 6, 12 and 18 (Fig. 1B). No significant increase of CPA was found at week 1 when compared to the control group (*p* = 0.9449). A significant increase of CPA was found from week 3 (2.71-fold, *p* = 0.0368) with a gradual and significant marked increase at week 6 (3.96-fold, *p* < 0.0002), week 12 (5.71-fold, *p* < 0.0001) and week 18 (8.34-fold, *p* < 0.0001). In HF-CDAA-treated mice, collagen deposition occurred in perisinusoidal regions from moderate (week 4), progressing to prominent periportal-sinusoidal fibrosis (week 8) and to bridging pan-lobular fibrosis with complete septa (week 12) (Fig. 2A)^6,23^. CPA was measured longitudinally at weeks 4, 8, 12 and 24: no significant increase of CPA was found at week 4 when compared to the control group (*p* = 0.7445). CPA greatly increased from week 8 (5.36-fold, *p* = 0.0166) up to week 12 (13.77-fold, *p* < 0.0001), with a stabilization of CPA at week 24 (12.98-fold, *p* < 0.0001). The reversibility of fibrosis and how it could be captured by automated CPA measurements were also investigated in this model. For this purpose, after 8 weeks of the HF-CDAA diet, mice were fed a regular diet for 1, 4 or 12 weeks (Fig. 2B). Histological observation of PSR-stained liver sections showed that switching HF-CDAA-treated mice to regular diet induced an obvious and substantial liver tissue remodeling characterized by a fragmentation of collagen bundles (Fig. 2A)^6^. The changes were observed in samples after 4 weeks of recovery and even more after 12 weeks. Despite these structural changes in fibrosis pattern, no statistically significant change of CPA was found between HF-CDAA treated mice at week 8 and those that had further recovered from injury for 1, 4 or 12 weeks (*p* = 0.8966; 0.1138; 0.9560, respectively), consistent with prior evidence that “recovery” from advanced fibrosis is not associated with substantial collagen removal (Fig. 2B)^20^.

### Automated CPA quantification enables rapid fibrosis assessment of entire liver sections at various magnifications

In CCl_4_ and HF-CDAA mouse models, different technical endpoints were studied for all WSI processed: analysis time, reproducibility, and impact of WSI magnification (20X: 0.452 µm/pixel and 40X: 0.226 µm/pixel). At 20X, CPA was determined within an average time of 12.5 s per section (n = 385 sections). Repeatability of automated CPA quantification was assessed by repeatedly running the software: a standard error of 0 ± 0 pixel was obtained over 5 reiterations, demonstrating 100% repeatability (data not shown). CPA values were equivalent whether computed on images scanned at either 20X or 40X magnification (Figs. 1C and 2C) for the two mouse models (r^2^ = 0.9989, *p* < 0.0001; r^2^ = 0.9985, *p* < 0.0001 for CCl4 and HF-CDAA, respectively).

### Automated CPA quantification is highly correlated with relative HYP content

**Figure 3:**
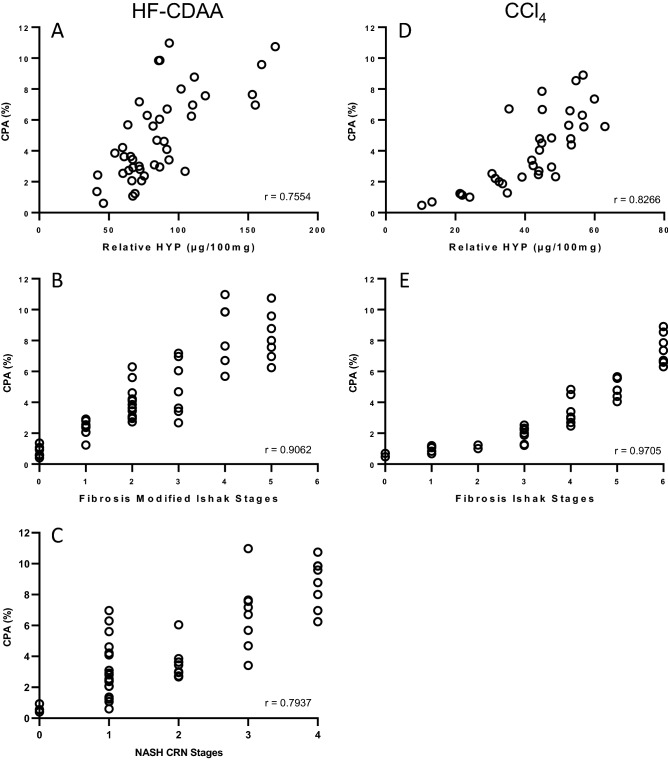
Correlation of CPA values with relative HYP content and fibrosis scores in HF-CDAA and CCl4 mouse models. The agreement of CPA values with those obtained with HYP content (**A** and **D**), fibrosis modified Ishak (**B**), Ishak (**E**) and NASH CRN (**C**) scores was evaluated using linear regression analysis. Data correspond to mean values per animal of control (n = 4–6 mice) and CCl_4_- or HF-CDAA-treated mice (6–10 mice/group). r = Spearman correlation coefficient. *p* value < 0.05 was considered statistically significant.

In CCl_4_ and HF-CDAA mouse models, liver fibrosis development was associated with a significant increase of the relative HYP content as shown before^6,20^. To validate automated CPA quantification as a reliable endpoint for the continuous measurement of liver fibrosis, CPA data were correlated with the corresponding relative HYP data obtained in the same mice. A high correlation was found for CCl_4_ model (r = 0.8266, *p* < 0.0001) and to a lesser extent for HF-CDAA model (r = 0.7554, *p* < 0.0001) (Fig. 3A and D).

### Automated CPA quantification is highly correlated with fibrosis staging

In clinics, liver fibrosis is currently graded using fibrosis staging systems such as the standardized NASH CRN^10^, or Ishak score^9^, depending on the underlying chronic liver disease. Correlations between automated CPA and fibrosis scores were determined to check whether liver fibrosis could be apprehended by CPA. Considering CCl_4_ mouse model, a high correlation was found between CPA data and Ishak stages (r = 0.9705, *p* < 0.0001) (Fig. 3E). In HF-CDAA-treated mice, fibrosis was staged using Ishak modified system as well as using the NASH CRN score, which is more relevant to the distribution of collagen fibers in this model (Table 1). High correlations were also found between CPA data and fibrosis modified Ishak stages (r = 0.9062, *p* < 0.0001) as well as with NASH CRN stages (r = 0.7937, *p* < 0.0001) (Fig. 3B,C).

**Table 1:** Fibrosis scoring systems used in the present study. NASH clinical research network (NASH CRN), Ishak and modified Ishak scoring systems definitions and scores used in the present study for determining the severity of liver fibrosis in CCl_4_ and HF-CDAA mouse models. NASH CRN scoring: from Kleiner et al., 2005^10^; Ishak scoring: from Ishak et al., 1995^9^.

NASH CRN system		Ishak scoring		Modified Ishak scoring	
Change	Score	Change	Score	Change	Score
No significant fibrosis	0	No fibrosis	0	No fibrosis	0
Mild zone 3 perisinusoidal fibrosis	1A	Fibrosis expansion of some portal areas, with or without short fibrous septa	1	Fibrous expansions of some central areas with or without short fibrosis septa	1
Moderate zone 3 perisinusoidal fibrosis	1B	Fibrous expansion of most portal areas, with or without short fibrous septa	2	Fibrous expansion of numerous central area with or without short fibrosis septa	2
Portal fibrosis only	1C	Fibrous expansion of most portal areas with occasional portal to portal bridging	3	Fibrous expansion of most central area with occasional fibrous bridge (centro-central or centro-portal)	3
Zone 3 perisinusoidal fibrosis with periportal fibrosis	2	Fibrous expansion of portal areas with marked bridging (portal to portal as well as portal to central)	4	Fibrous expansion of central area with marked bridging (centro-central, centro-portal)	4
Bridging fibrosis	3	Marked bridging (portal to portal or portal to central) with occasional nodules	5	Marked bridging with occasional nodule	5
Cirrhosis	4	Cirrhosis, probable or definite	6	Cirrhosis (probable or definitive) (nodular dissection)	6

### Automated CPA quantification correlates better with fibrosis stages than HYP assay

HYP assay has been the gold standard for the quantification of liver fibrosis in rodents for many years. At bedside, liver fibrosis cannot be evaluated by HYP assay which requires a large amount of tissue. The reference in the clinic is the histopathological staging score from liver biopsy. Therefore, since preclinical investigations are conducted with the aim of mimicking human disease, we thought it was essential to validate the potential value of CPA by comparing quantitative CPA and HYP analyses to the staging systems as reference (Table 2). For both CCl_4_ and HF-CDAA mouse models, CPA better correlated with fibrosis stages than HYP assay, regardless of the score used (*p* < 0.0001 for Ishak on CCl4 livers; *p* = 0.010 and *p* = 0.031 for modified Ishak and NASH CRN for HF-CDAA group, respectively).

**Table 2:** Comparison of Spearman correlation rates performed with MorphoQuant CPA quantitative analysis and hydroxyproline (HYP) assay using the one-sided Fisher’s Z transformation, considering histopathological scoring as the reference.

Mouse model	Staging score	Spearman r with CPA	Spearman r with HYP	Z-score (CPA vs HYP)	*p*-value (one-sided)
CCl4 (n = 35)	Ishak	0.96 [0.928–0.982]	0.81 [0.648–0.902]	4.786	< 0.0001*
HF-CDAA (n = 43)	Modified Ishak	0.89 [0.795–0.938]	0.76 [0.583–0.863]	2.316	0.0102*
	Fibrosis CRN	0.75 [0.570–0.858]	0.59 [0.337–0.757]	1.861	0.0314*

## Discussion

The automated image analysis software investigated in the present study demonstrated that accurate, reliable, reproducible and rapid quantification of liver fibrosis can be assessed from entire PSR-stained liver sections at high magnification, and independently from experimenter’s intervention. The automated analysis of liver fibrosis was based on the continuous measurement of the collagen proportionate area (CPA)^13,14^. Other studies in rodents^15,24–26^ and human biopsies^26–28^ used CPA to report on the amount of collagen deposit in the liver. In these previous studies, CPA measurement still involved a number of manual procedures, for example to select relevant area or exclude artefacts, unlike the present study in which it was fully automated from native images to statistical analysis. This provides a tremendous benefit since it is totally operator-independent, eliminating possible intra- and inter-variability in collagen quantification. In addition, automation significantly reduces analysis time paving the way for high throughput analysis of liver histological sections as demonstrated in the present study. It is noteworthy that the fibrosis measurement was carried out on the entire liver section, at high magnification. Another important breakthrough of the present automated image analysis is that detection and quantification criteria used were strictly identical for all liver sections analyzed which allows valid comparison and standardization.

In most rodent studies, quantification of liver fibrosis refers to the biochemical analysis of HYP content^6,15,23^ For the purpose to validate the quantification of collagen content with digital quantification of collagen, the correlation with HYP content was investigated in both CCl_4_ and HF-CDAA mouse models. It should be noted that relative HYP content was determined from a large volume of liver tissue (250–300 mg) representing $\sim$ 20% of the whole liver which may therefore better reflect scar collagen deposition throughout the liver^21^, essentially overcoming the sampling error. Surprisingly, although CPA analysis was performed on a small volume of tissue (3 entire sections/mouse), the results indicated that CPA and relative HYP content were highly correlated in both fibrotic mouse models. Such a correlation strongly suggests that in liver fibrosis mouse models the CPA remarkably reflects the extent of fibrosis. Ultimately, the high correlation found between CPA and HYP contributes to validate CPA index as an endpoint measurement of liver fibrosis which, when combined with the HYP assay, leads to a better overall assessment of liver fibrosis.

The automated analysis of liver fibrosis based on the measurement of the CPA was highly correlated with Ishak score in CCl_4_ model as well as with NASH CRN and the modified Ishak scores in HF-CDAA model, both assessed on the same PSR-stained sections. Correlations between fibrosis scores and CPA were previously investigated in other fibrotic rodent models^8,15,29^ and human liver biopsy^17,28,30,31^ contributing to validate CPA as a direct readout of changes of collagen deposit in liver tissue. In CCl_4_ model, we showed that CPA highly correlated with Ishak score (r = 0.9705). In HF-CDAA model, CPA better correlated with the modified Ishak score (r = 0.9062) than with NASH CRN (r = 0.7937) system. One would expect collagen measurement to be in better agreement with the NASH CRN since this latter was specifically developed for fibrosis with underlying NASH. This difference is likely related to the fact that Ishak score may be more precise given its greater number of scores (n = 6) compared to those (n = 4) of NASH CRN system. We have also shown that correlations between CPA and scoring systems were higher than those observed between scores and HYP. This may be due to the fact that CPA quantification and scoring analysis were performed on the same histological liver sections. However, fibrosis assessed through CPA brings a new dimension by providing quantitative data, on top of fibrosis stage and localization.

Interestingly, correlations we found between CPA and scoring systems or HYP were consistently higher for the CCl_4_ mouse model than that for the HF-CDAA. We have to point out that CCl_4_ and HF-CDAA models differ dramatically from each other in their liver fibrosis pattern, which is mostly septal in CCl_4_ model (Fig. 1A) and mostly perisinusoidal, “chicken wire” in HF-CDAA model (Fig. 2A). It appears highly plausible that discrepancies we found between CPA and HYP collagen assessment in these mouse models might be linked to disparity in PSR staining of collagen fibers according to their septal or perisinusoidal location. HYP assay principle is based on determination of collagen-specific amino-acid after exhaustive tissue hydrolysis and should therefore capture all collagen fibers regardless of their lobular location or maturation state. CPA, on the other hand, depends on detection of picrosirius chromogen that intercalates with triple helical collagen and, in theory, may be more challenging to visualize freshly deposited, immature collagens that are not yet organized into thick septae and that appear to dominate the scar in HF-CDAA model compared to CCl4 model (Figs. 1A and 2A). One apparent conclusion can be that while histological methods are standard and most commonly used, only different and complementary methodological approaches allow an in-depth capture of the entirety of fibrotic phenotype across different rodent models.

In human pathology, the scoring systems describe fibrosis pattern and distribution and provide clinically relevant information driving patient’s prognostic and management. The small number of stages is a major obstacle to describe the kinetics of fibrosis during disease progression and to assess the response to an intervention therapy, especially in clinical trials in which signs of efficacy are needed early in comparison to the timing for fibrosis remodeling or natural history. On the other hand, having quantitative data on fibrosis amount is not sufficient to correctly address patients, as localization drives prognosis. It is our opinion that both readouts, fibrosis localization and amount, are needed to better capture fibrosis status, and understand its dynamics. Therefore, CPA appears has a cardinal complementary assessment to the standard histopathological evaluation of liver fibrosis, delivering an objective continuous quantitative assessment of the amount of fibrosis, and the scores providing data on fibrosis distribution.

In conclusion, the automated computerized image analysis applied in the present study provides an accurate, reliable and fast continuous quantification of liver fibrosis induced in translational mouse models. The automation of the present image analysis is a major breakthrough since it provides a fully experimenter-independent measurement of liver fibrosis. Other investigations are in progress to carry out automated fibrosis quantification in relation to its specific localization i.e., perisinusoidal, perivascular or septal. Since the present image analysis proves to represent a powerful tool for the evaluation of fibrosis in preclinical models, its implementation for the monitoring of fibrosis evolution on human biopsies is currently being investigated.

## Materials and methods

### Animals

Seven-weeks old male C57Bl/6 J mice were obtained from Jackson Laboratory, Bar Harbor, ME and housed in a temperature- and humidity-controlled environment in a 12-h light–dark cycle with water and standard mouse pellet chow ad libitum. After acclimatization for one week, mice were either fed with a HF-CDAA diet or chemically treated with CCl_4_ to induce liver fibrosis.

HF-CDAA diet manufactured by Research Diets (New Brunswick, NJ) was supplemented with high fat (60% kcal as fat, A06071323) and applied for 4, 8, 12 and 24 weeks. In order to investigate fibrosis reversibility in HF-CDAA mouse model, some mice (n = 13) were switched to a regular chow after 8 weeks of HF-CDAA diet. Four C57Bl/6 J mice fed with regular chow for 8 weeks were used as normal controls for HF-CDAA mice group.

CCl_4_ treatment was performed via oral gavage (dissolved in mineral oil) thrice a week and applied for 4, 8, 12 or 18 weeks according to an escalating dose protocol optimized for C57Bl6J strain described before^20^. Four C57Bl/6 J mice treated with mineral oil were used as normal controls for CCl_4_-induced fibrotic mice.

At defined time points reflecting disease progression, mice were sacrificed by an overdose of ketamine/xylazine anesthesia and livers were excised. Liver specimens from two lobes were either fixed in 4% buffered formalin or snap-frozen in liquid nitrogen for further analysis. All animal experiments were performed in compliance with the relevant laws and institutional guidelines and in accordance with the ARRIVE guidelines. They were approved by the Institutional Animal Care and Use Committee (Beth Israel Deaconess Medical Center BIDMC, protocols 158-2008 and 004-2012) and conducted in the same facility room at BIDMC.

### Hepatic hydroxyproline determination

Relative HYP content (µg/g liver) was used as a surrogate marker for hepatic collagen content and determined biochemically as described before^32^. Two freshly harvested liver pieces (one from the left and one from the right lobe) amounting to 250 to 300 mg in total (representing > 10% of whole liver) were hydrolyzed in 6 N HCl for 16 h at 110 °C, followed by calorimetric determination of hydroxyproline^33^.

### Liver histology and fibrosis staging

Liver samples from two different lobes (harvested similarly to hydroxyproline samples in standardized fashion) were formalin-fixed and mounted in paraffin-embedded blocks. Three 5-µm sections apart from 50 µm each per animal were used and stained with PSR as described before^14^. PSR-stained liver sections were then scanned at 20x (0.452 µm/pixel) and 40x (0.226 µm/pixel) using a NanoZoomer-SQ and their whole slide image (WSI) captured using the NDP.view 2 software (both from Hamamatsu Corporation, Japan).

WSI were scored blindly for fibrosis according to the NASH CRN System^10^ and/or Ishak score^9^ by a hepatologist (IL) trained to histological assessment of liver diseases in preclinical rodent models. CCl_4_ causes centrilobular injury and thus fibrosis initiates in pericentral zones. Therefore, the Ishak Scoring system, developed for characterization of fibrosis that readily initiates in periportal area in human liver diseases, was modified accordingly to better fit the evolution of the fibrosis caused by repeated exposure to CCl_4_ (Table 1). Whatever the scoring system used, the three sections per animal were scored, and the highest individual unit value was used for further statistical analysis.

### Automated quantification of liver fibrosis

The automated quantification of liver fibrosis was carried out by means of MorphoQuant software (Biocellvia, France) from the same WSI used presently for histological scoring. Tissue area of the entire liver section was automatically delineated followed by an accurate segmentation of the PSR-stained collagen fibers as described before^13,14^. Briefly, the optical density (absorbance) is computed on each channel of the submitted image to match the human perception as closely as possible. A single value decomposition (SVD) is then performed on the resulting image, and each pixel is projected along the third singular vector, resulting in a one-dimensional image. Finally, a simple threshold is used to get only the red pixels from the original image. Liver fibrosis was expressed as collagen proportionate area (CPA) corresponding to the ratio of the area of PSR-stained collagen fibers to the area of the entire liver section. The analysis was conducted at the two different high magnifications, 20X and 40X, to investigate the impact of the magnification on the software performances. The reproducibility of the automated quantification of liver fibrosis was tested on the whole set of slides by repeatedly running the software 5 times.

### Statistical analysis

Data were expressed as mean ± standard deviation. For CPA analysis, a mean per subject was calculated from the 3 sections. For the longitudinal analysis of liver fibrosis in the two investigated models, statistical differences between groups were analyzed using a one-way ANOVA followed by Dunnett’s multiple comparison post-hoc test (GraphPad Prism 9.0; GraphPad Software, Inc. La Jolla, CA). Linear regression analysis was then performed to compare CPA assessment at 20X and 40X magnifications. Statistical analysis was performed using the non-parametric Spearman correlation coefficient to estimate the correlations between automated CPA and HYP assay on one hand, and CPA and fibrosis scores on the other hand. A *p*-value < 0.05 was considered statistically significant. To further characterize the automated CPA quantification as an endpoint measurement of liver fibrosis, correlation rates of histopathological scoring with CPA or HYP were compared using the one-sided Fisher’s Z transformation, considering histopathological scoring data as references^34^. A *p*-value < 0.05 was considered statistically significant.

## Data Availability

The datasets used and/or analyzed during the current study are available from the corresponding author on reasonable request.
